# Field-driven domain wall motion under a bias current in the creep and flow regimes in Pt/[CoSiB/Pt]_*N*_ nanowires

**DOI:** 10.1038/srep23933

**Published:** 2016-03-31

**Authors:** Y. H. Choi, Y. Yoshimura, K.-J. Kim, K. Lee, T. W. Kim, T. Ono, C.-Y. You, M. H. Jung

**Affiliations:** 1 Department of Physics, Sogang University, Seoul 121-742 Korea; 2 Institute for Chemical Research, Kyoto University, Uji, Kyoto 611-0011, Japan; 3 Institute of Physics, Johannes Gutenberg-Universität Mainz, 55128 Mainz, Germany; 4 Department of Advanced Materials Engineering, Sejong University, Seoul 143-747 Korea; 5 Department of Physics, Inha University, Incheon 402-751, Korea

## Abstract

The dynamics of magnetic domain wall (DW) in perpendicular magnetic anisotropy Pt/[CoSiB/Pt]_*N*_ nanowires was studied by measuring the DW velocity under a magnetic field (*H*) and an electric current (*J*) in two extreme regimes of DW creep and flow. Two important findings are addressed. One is that the field-driven DW velocity increases with increasing *N* in the flow regime, whereas the trend is inverted in the creep regime. The other is that the sign of spin current-induced effective field is gradually reversed with increasing *N* in both DW creep and flow regimes. To reveal the underlying mechanism of new findings, we performed further experiment and micromagnetic simulation, from which we found that the observed phenomena can be explained by the combined effect of the DW anisotropy, Dzyaloshinskii-Moriya interaction, spin-Hall effect, and spin-transfer torques. Our results shed light on the mechanism of DW dynamics in novel amorphous PMA nanowires, so that this work may open a path to utilize the amorphous PMA in emerging DW-based spintronic devices.

Materials with perpendicular magnetic anisotropy (PMA) accelerates the developments of spintronics devices due to their low threshold current density, simple domain wall (DW) structure, and high spin-transfer-torque (STT) efficiency, compared with in-plane magnetic anisotropy (IMA) materials^1^,^2^,^3^. They can be used as one of magnetic components in magnetic memory and logic devices by manipulating the magnetic domain wall (DW) motion^4^,^5^,^6^. Thus, it is important to understand the DW dynamics in PMA materials. There have been some experimental and theoretical reports to study the DW motion driven by magnetic field^7^,^8^,^9^,^10^,^11^,^12^,^13^. However, most of the studies have been done with polycrystalline permalloy with IMA^14^,^15^,^16^ and Co/Pt and Co/Ni superlattices with PMA^17^,^18^,^19^,^20^. Recently, amorphous Pt/[CoSiB/Pt]_N_ multilayers have been reported to have strong PMA with high squareness. Owing to the strong PMA and low pinning coming from the amorphous nature, they are expected to have thinner DW width and smoother DW motion than in crystalline PMA multilayers^21^,^22^. Therefore, the amorphous Pt/[CoSiB/Pt]_N_ multilayers are good candidates to study the role of spin-transfer torque (STT), spin-orbit related phenomena including spin Hall effect (SHE) and Rashba effect (RE), and Dzyaloshinskii-Moriya interaction (DMI).

In this study, we investigate the field-driven DW motion with positive and negative constant currents on Ta(50 Å)/Pt(14 Å)/[CoSiB(6 Å)/Pt(14 Å)]_N_ nanowires for various *N* = 3, 6 and 9 and widths *w* = 150, 300 and 500 nm. Two important findings are addressed by exploring the DW motion in two extreme regimes of DW creep and DW flow. First, the field-driven DW velocity is found to increase with increasing *N* in DW flow regime, whereas it is inverted in the DW creep regime. Second, the sign of spin current-induced effective field is gradually reversed with increasing *N*. For the intermediate *N*, the sign change of spin current-induced effective field is also observed with increasing magnetic field, implying that the role of spin current depends on the dynamic regime of DW. To reveal the underlying mechanism of the observed phenomena, we check the all known possible mechanisms such as DW anisotropy, DMI, SHE, and STT. The DW anisotropy is calculated and found to gradually increase with *N*. Since the higher the DW anisotropy, the faster the DW velocity in the precessional flow regime, the trend of the DW anisotropy can account for our first finding. Such an enhancement of DW anisotropy also suggests that spin waves can be emitted during the DW motion which also accelerates the DW motion^23^. The DMI, which may affect both the field- and spin current-driven DW motion, is experimentally determined by measuring the DW velocity with applying an in-plane bias field. We find that there exists finite DMI in our samples, but the sign as well as strength of DMI does not significantly depend on *N*, manifesting that the DMI does not play the main role in the observed phenomena. The SHE and STT effects are calculated based on the reported material parameters, and it is found that their relative strength gradually changes with increasing *N* from SHE dominant for low *N* to STT dominant for high *N*. This can explain our second finding qualitatively, because the SHE (STT) assists the DW motion along the current (electron) flow direction.

## Results and Discussion

**Figure 1:**
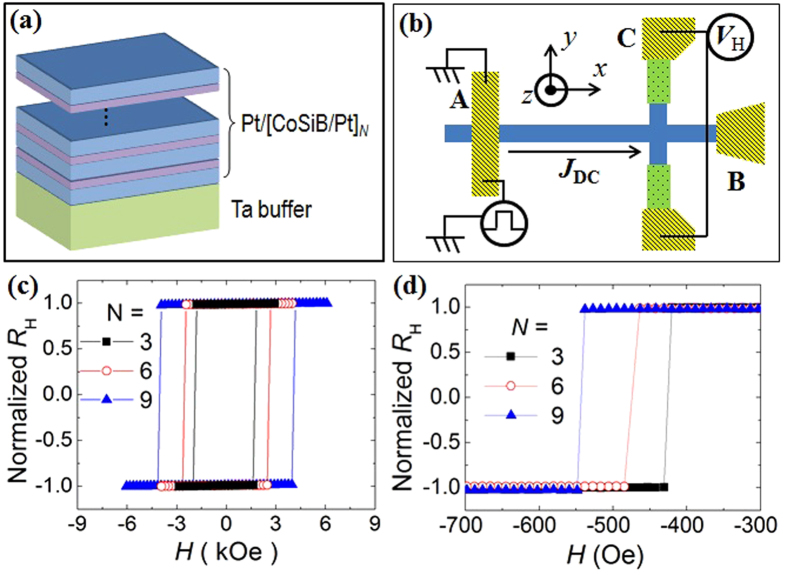
Sample characterizations. (**a**) Sample structure and (**b**) schematic illustration of the experiment setup of [CoSiB/Pt]_N_ nanowires. A is the electrode to inject a pulse current and create a DW. DC current is applied into A and B electrodes and the Hall voltage is measured at C electrode. (**c**) Normalized Hall resistance *R*_*H*_ data of [CoSiB/Pt]_N_ nanowires for *N* = 3, 6 and 9. The switching field corresponds to the coercive field. (**d**) Normalized Hall resistance *R*_*H*_ data after creating DW when perpendicular magnetic field pushes the DW to Hall bar. The switching field corresponds to the depinning field.

Thin films of Pt(14 Å)/[Co_75_Si_15_B_10_(6 Å)/Pt(14 Å)/]_N_ multilayers (*N* = 3, 6 and 9) were prepared on Si substrates with buffer layers of Ta(50 Å) using a dc magnetron sputtering system at room temperature^22^. Figure 1(a) shows the schematic structure of the samples. We note that the CoSiB layers have symmetric interfaces because they are sandwiched between two nominally identical Pt layers, that is Pt/[CoSiB/Pt]_N_. The only structural inversion asymmetry comes from the bottom Ta layer. The magnetization measurements revealed that the effective anisotropy constant *K*_1_^eff^ and the saturation magnetization *M*_*S*_ are 1.5 × 10^5^ J/m^3^ and 3.5 × 10^5^ A/m, respectively. *K*_1_^eff^ and *M*_*S*_ are not significantly altered by the layer thickness^21^. These PMA films were patterned into nanowire devices with Hall bar structure using electron beam lithography and ion milling. The width of nanowires ranged from *w* = 150, 300 to 500 nm and the length was fixed to be *L* = 50 μm. Figure 1(b) shows the schematic illustration with the experimental set-up for the DW motion measurements. There are two electrodes of A and B at the ends of nanowires to inject a dc current and create a DW, and there is one more electrode of C at the Hall cross bar to measure the anomalous Hall voltage. Once the DW moves from A to B, the Hall voltage, *V*_*H*_, is detected because it is proportional to the perpendicular component of magnetization and is much larger than the ordinary Hall voltage in ferromagnetic materials. In Fig. 1(c), the measured normalized Hall resistance curves are plotted as a function of the external magnetic field *H* applied perpendicular to the film plane, for *N* = 3, 6 and 9 with the width of 300 nm at room temperature. Hysteresis loops are clearly observed with different coercive fields *H*_*C*_ = 1.8, 2.5 and 4.0 kOe for *N* = 3, 6 and 9, respectively. This enhancement of *H*_*C*_ with *N* in the nanowires is similar to that observed in the un-patterned films, but the *H*_*C*_ values are much larger^22^. In addition to *H*_*C*_, it is also important to determine the depinning field *H*_*dep*_ for understanding the DW dynamics. First, we applied a strong positive perpendicular magnetic field (+*H*_*z*_ = 4 kOe) into the entire nanowire to saturate the magnetization of nanowire, and we injected a pulsed current (80 mA, 15 ns) into the electrode A to create DW by using the local Oersted field. Then, we swept the magnetic field along the negative *z*-direction, and finally we detected the Hall voltage *V*_*H*_ at the electrode C at a constant dc current (0.02 mA). As shown in Fig. 1(d), a clear switching was observed in the Hall resistance *R*_*H*_ data when the DW passed through the Hall cross. This switching field is the DW depinning field *H*_*dep*_, which increases with increasing *N* from 430, 485 and 550 Oe for *N* = 3, 6 and 9, respectively. The depinning effect depends on the strength of pinning site in the material. Note that *H*_*dep*_ is always lower than *H*_*C*_ of the nanowire.

**Figure 2:**
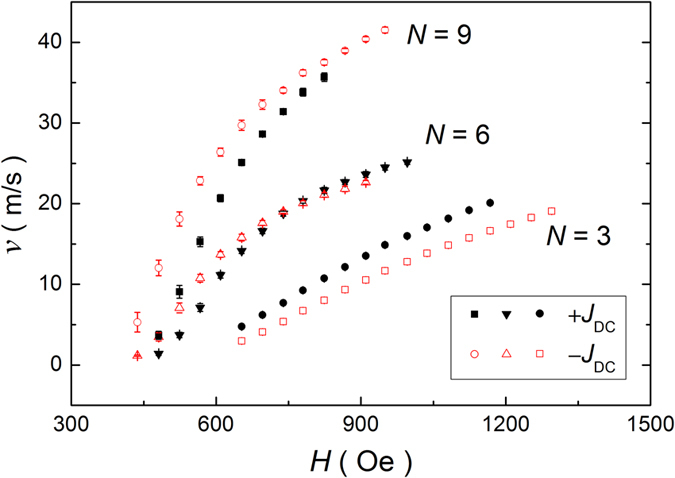
DW velocity *v* versus magnetic field *H*. The DW velocity was taken by real-time measurements for [CoSiB/Pt]_N_ nanowires with different number of layers *N* (=3, 6 and 9) and wire width *w* = 500 nm. The closed and open symbols indicate the DW velocities measured in positive and negative dc current density *J*_*DC*_ = ±5.4 × 10^11^ A/m^2^, respectively. The data were plotted by averaging the measurements ~100 times.

Figure 2 represents DW velocities in the flow regime taken with ±*J*_*DC*_ for various *N* and *w* = 500 nm. The first main finding is that the field-driven DW velocity increases with *N*. Considering that material parameters that can affect the DW velocity do not significantly depend on *N*, another influential factor should be taken into account to explain the experimental results. The second finding is that there is a big difference on the role of spin current in DW motion with increasing *N*: for *N* = 3, the DW velocity measured at +*J*_*DC*_ is faster than that measured at −*J*_*DC*_, while for *N* = 9, the DW velocity at *−J*_*DC*_ is faster than that at +*J*_*DC*_. This means that conventional bulk STT cannot explain the experimental results, because it should be independent on *N*. Thus, interfacial effects should be considered to account for the observed results.

**Figure 3:**
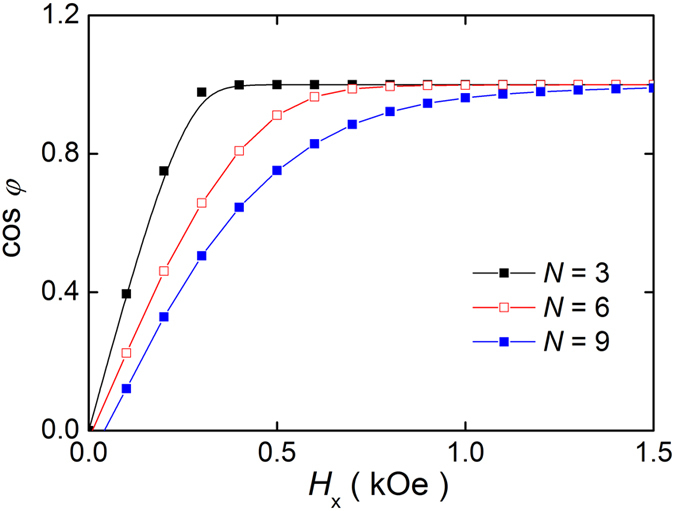
Equilibrium DW angle *φ* as a function of longitudinal in-plane magnetic field *H*_x_. The DW angle was obtained for different *N* from the micromagnetic simulation. Here, cos *φ* = 0 (cos *φ* = 1) corresponds to the Bloch DW (Néel DW).

Let us discuss the first finding, i.e. increment of the DW velocity with *N*. Figure 2 shows that the DW velocity for *N* = 6 and 9 are faster than that for *N* = 3 by a factor 2 and 4 at the same external field. To reveal the underlying mechanism of such a large increase of DW velocity with *N*, we compare the experimental results with the well-established DW motion theory. The DW velocity in the flow regime is described by the Walker model^24^, in which the DW velocity increases linearly with the external magnetic field up to a threshold field (hereafter the Walker field *H*_W_), beyond which it abruptly decreases. As the magnetic field increases further, the DW velocity again slightly increases with the magnetic field. This unique nonlinear feature of DW velocity is due to the change in the dynamic mode of DW from steady motion below *H*_W_ to precessional motion beyond *H*_W_, which is referred to as the Walker breakdown (WB) phenomenon. In each dynamic regime, the DW velocity is described by


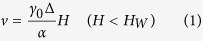



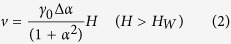


where *γ*_0_ is the gyromagnetic ratio, Δ the DW width, and *α* the Gilbert damping parameter. It is thus important to distinguish *H*_W_ to understand the observed DW velocity. The *H*_W_ is described by^25^


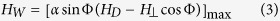


where Φ is the angle of DW magnetic moment, *H*_*D*_ the DMI-induced effective field as we discuss later, and *H*_⊥_ the DW anisotropy field that corresponds to the energy difference between the Bloch and Néel DWs. We estimate *H*_⊥_ for different *N* by the micromagnetic simulation^26^. Figure 3 shows the equilibrium DW azimuthal angle *φ* as a function of the in-plane longitudinal magnetic field (*H*_x_) for different *N*. The sample having larger *N* requires larger *H*_x_ to make Néel DW configuration, indicating that *H*_⊥_ gradually increases with *N*. Such an enhancement of *H*_⊥_ with *N* may originate from the variation of the demagnetizing factor depending on the geometry. From the estimated *H*_*D*_ and *H*_⊥_, the *H*_W_ is found to be about 174, 262 and 337 Oe for *N* = 3, 6 and 9 by assuming *α* = 0.5. Obviously, the *H*_W_ is found to be smaller than the depinning field (Fig. 1(d)), implying that the WB phenomenon is obscured by the pinning in the wire. This is the reason why we did not observe any velocity breakdown in Fig. 2. The estimation of *H*_W_ also gives us an important fact that the DW velocities observed in Fig. 2 are come from the precessional DW motion. Thus, Eq. (2) more likely explains the observed DW velocity.

However, all variables in Eq. (2) are not changed with *N* because they are all the intrinsic material parameters^21^, and Eq. (2) is satisfied in a limit case of very high fields. However, our experimental conditions are moderate case, so that Eq. (2) cannot properly describe our experimental results. Therefore, we need more deep understanding to explain the first findings. Here we present two possible origins of our observation. The first scenario is that the observed velocity lies in-between the peak velocity at *H*_W_ and fully linear precessional regime, where the abrupt reduction and gradual increase of DW velocity are predicted. In this intermediate regime, the DW velocity is mainly determined by the peak velocity at *H*_W_. Note that *H*_W_ for *N* = 6 and 9 are larger than that for *N* = 3 by a factor of 2 and 3 and the peak velocity should have the same trend with *H*_W_. Thus, the increment of peak velocity can qualitatively explain the velocity enhancement with *N*. The measured field in our experimental range is not far away from the *H*_W_ (see Supplementary Information), which signals that the measurement range lies on the intermediate regime. The second possible origin is the emission of spin waves. As discussed in ref. 23, a large DW anisotropy causes a spin wave emission during the turbulent DW motion. Such a spin wave emission can be considered as an additional damping, and thus it accelerates the DW velocity^15^,^23^. Considering that the DW anisotropy increases with *N*, spin wave emission can properly explains the DW velocity enhancement with *N*. Two remarks are in order. First, because of the non-negligible degree of freedom along the wire transverse direction, two-dimensional aspect of DW may affect the DW velocity. In two-dimensional DW, precessional DW motion is replaced by the evolution of vertical Bloch lines inside the DW. However, the overall velocity versus field curve for two-dimensional wires is known to be not much different from that for one-dimensional wire. Thus, the formation of the vertical Bloch lines may not significantly affect the velocity enhancement with *N*. Second, it has been recently reported that DMI also affect the DW velocity by changing *H*_W_ and corresponding peak velocity^27^. However, we found that the DMI cannot explain the result in Fig. 2, since it has opposite trend with *N*, as discussed later.

Before we discuss more detail on the second finding, i.e., the transition in the current dependency of the DW motion with *N*, let us consider the possible sources of DW motion in our experiments. According to the structure of our samples, Ta(50 Å)/Pt(14 Å)/[CoSiB(6 Å)/Pt(14 Å) ]_*N*_, the magnetic CoSiB layer is sandwiched by the nominally identical Pt layers, so that RE, SHE, and DMI arising from the internal Pt layers are small or vanished because of the inversion symmetry. The structural gradient, possibly originating from different interfacial properties between Pt/CoSiB and CoSiB/Pt, can generate RE and DMI in our system, which was recently evidenced in textured Co/Pd multilayer multilayer^28^ or ordered FePd L1_0_ structure^29^. Therefore, we will consider contribution of SHE, RE and DMI in our observations.

**Figure 4:**
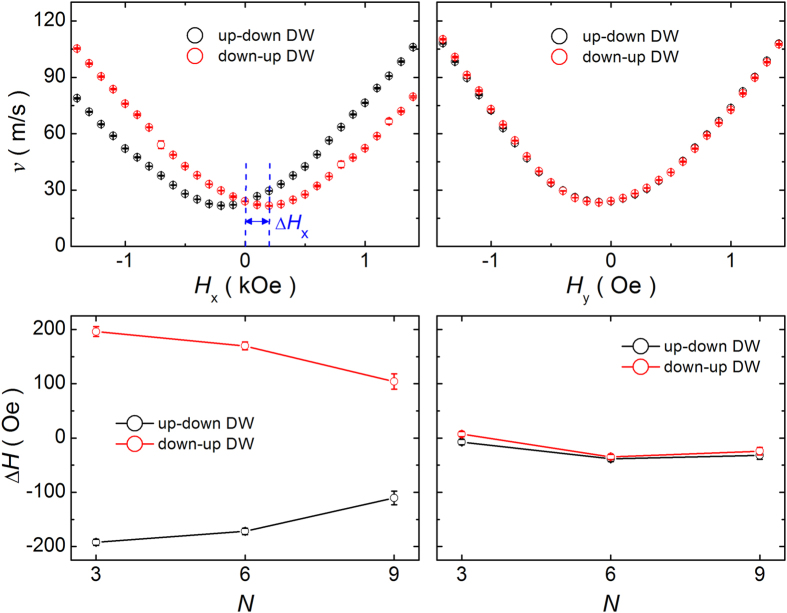
DW velocity *v* as a function of longitudinal and transverse in-plane magnetic field *H*_x_ and *H*_y_. The DW velocity was taken in the flow regime for [CoSiB/Pt]_N_ nanowires with varying *N* (=3, 6 and 9) and *w* = 1 μm. Each experimental data point was taken by averaging the measurements ~10 times.

We first checked the existence of DMI in our samples. Figure 4(a,b) shows the DW velocity in the flow regime as a function of longitudinal and transverse in-plane magnetic field *H*_x_ and *H*_y_ for *N* = 6. Here, we fixed the out-of-plane magnetic field as |*H*_*z*_| = 800 Oe and varied the in-plane magnetic field. The DW velocity shows clear asymmetry for the in-plane longitudinal field (*H*_x_), while it exhibits a symmetric behavior for the in-plane transverse field (*H*_y_). Furthermore, the sign of asymmetry in the DW velocity versus *H*_x_ reverses when the DW type is changed from the up-down to the down-up DW. This implies that the homochiral DW is formed in our samples possibly due to the non-negligible interfacial DMI effect which may come from the dissimilar interfaces between Pt/CoSiB and CoSiB/Pt^30^. The strength of DMI can be quantified by estimating the horizontal shift in the DW velocity versus *H*_x_, which corresponds to the DMI-induced effective field *H*_DMI_. In Fig. 4(c,d), we summarize the horizontal shift field in the DW velocity versus *H*_x_ (or *H*_y_) with respect to *N*. It is clear from Fig. 4(c) that there exists finite DMI in our samples and the strength of DMI decreases with increasing *N*. This suggests that DMI is not the origin of our first finding, i.e., the DW velocity enhancement with increasing *N*, since the higher DW velocity requires higher DMI^27^,^31^.

The finite value of DMI suggests a possibility to explain our second finding, i.e, the transition in the current direction dependency of the DW motion with *N*, because the existence of DMI supports the action of SHE on the DW motion. Let us consider the role of SHE in the ferromagnetic layer with heavy metals,^29^. The SHE in heavy metal generates a spin current which is injected into the ferromagnetic layer. Such a spin current exerts a torque to the magnetic moment of ferromagnetic layer, which can be described by^32^,^33^,^34^


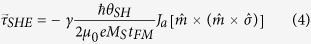


where *γ* is the gyromagnetic ratio, 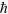 the Plank constant, *θ*_*SH*_ the spin Hall angle, *μ*_0_ the vacuum permeability, *e* the electron charge, *M*_*S*_ the saturation magnetization, *t*_*FM*_ the thickness of ferromagnet, *J*_*a*_ the applied current density, 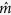 and 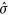 are the unit vectors of magnetic moment and injected spin. Equation 4 implies that the SH torque deviates the magnetic moment from the stabilized direction which is determined by the DW anisotropy and the DMI. Then, the restoring field induced by the DW anisotropy and the DMI immediately rotates the magnetic moment, which is the main DW driving mechanism. The SH torque itself also drives the DW when the Gilbert damping is not negligible. In this case, we can easily understand the effect of SH torque as an effective field such as 
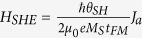
. Therefore, the SHE, if it exists, can affect the DW motion. The effect of SH torque on the precessional DW motion is discussed in Supplementary Information.

Let us next discuss the source of spin current in our device. Two possible sources can be considered. First, two Pt layers located in the bottom or in the top of CoSiB layer may not be identical to each other, which causes a non-uniform current flow inside the multilayer structure. Indeed, such a non-uniform current flow was recently reported in a similar multilayer structure, where the authors claimed that the current flowing along the top Pt layer is larger than that flowing along the bottom Pt layer^35^. We also would like to note that the finite DMI in our sample also supports such dissimilarity between top and bottom Pt layers. Second possible source of spin current is the bottom Ta layer since the Ta is the only part that the structural symmetry is broken and the spin diffusion length of Pt is longer than its thickness^36^. Thus, we cannot rule out the contribution of spin current from Ta layer. We note that any source of spin current, i.e, either bottom Ta or top Pt, can move the DW to the same direction because the Ta and Pt are well known heavy metals with opposite sign of spin Hall angle. In addition, if we consider the sign of DMI in our sample that induces a right handed chiral DW, the SHE effect pushes the DW to the current flow direction.

Since STT and SOT are orthogonal to each other, we cannot directly compare them but we simply estimate their relative contributions to the DW motion. The STT can be expressed by the velocity unit 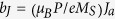, where *P* is the spin polarization and *μ*_B_ the Bohr magneton. Considering that the *P* value of Co layer in Co/Pt multilayer is less than 1%^36^, the *P* value of CoSiB layer in CoSiB/Pt multilayer must be smaller than 1%. Then, *b*_*J*_ becomes small (~1 m/s) in our experiments, and thereby the SHE becomes rather significant. Since the SHE is mostly significant for thin *t*_*FM*_ case (*N* = 3), the DW is slower in −*J*_*DC*_ than +*J*_*DC*_. However, for thicker *t*_*FM*_ case (*N* = 9), the SHE is less significant because of its inverse proportionality to *t*_*FM*_, so that the DW is faster in −*J*_*DC*_ than +*J*_*DC*_. This simple explanation can explain our second finding; the DW motion changes the preferred direction from current flow to electron flow with increasing *N*. It is worthwhile to note that the observed transition of current dependency is not exclusively linked to the SHE but may arise also due to other sources of spin accumulation, such as the Rashba effect^37^.

**Figure 5:**
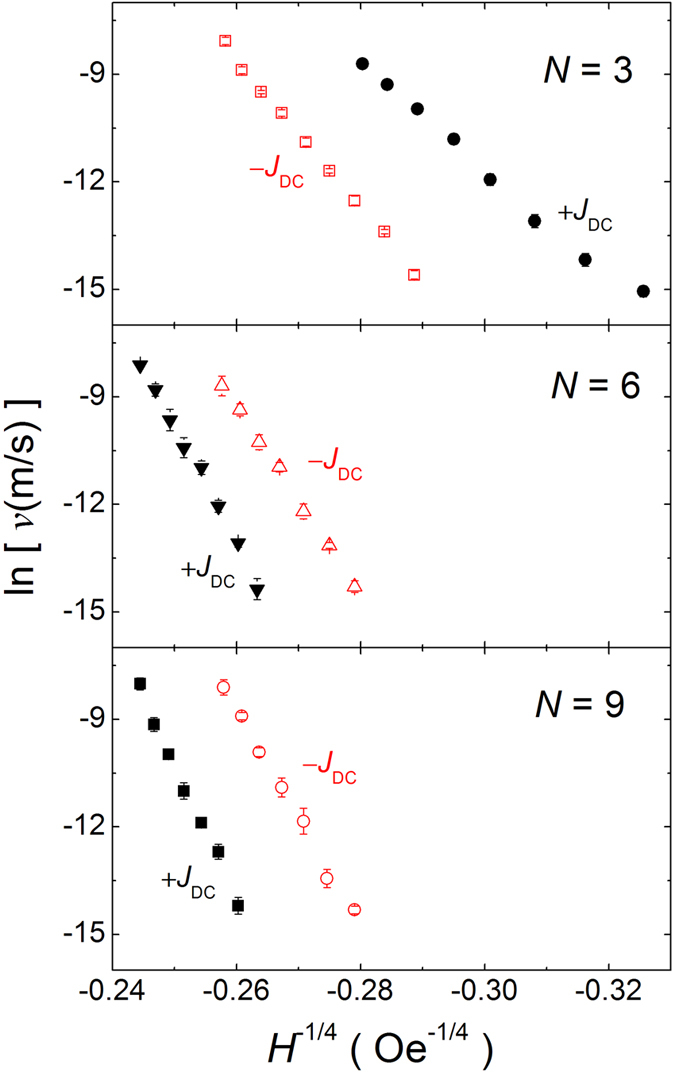
DW velocity *v* as a function of H^−1/4^. The DW velocity was taken in the creep regime for [CoSiB/Pt]_N_ nanowires with varying *N* (=3, 6 and 9) and *w* = 500 nm. Each experimental data point was taken by averaging the measurements ~500 times.

Interestingly, the effect of spin current on the DW velocity gradually changes as increasing magnetic field for *N* = 6. At low fields, the DW velocity is faster in −*J*_*DC*_. On the other hand, the DW velocity becomes faster in +*J*_*DC*_ at high fields. This implies that the effect of spin current is different depending on the DW dynamic regime, because the DW creep motion emerges in a small field regime^17^. Thereby, we checked the DW motion in the creep regime. In Fig. 5, the logarithmic DW velocities are plotted as a function of *H*^−1/4 ^17. The DW velocity in the creep regime can be described by^38^.


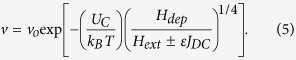


Here, the exponent of −1/4 is a fingerprint of the two-dimensional DW motion^22^, *U*_*C*_ the pinning potential barrier, and *ε* the current-to-field efficiency that is induced by either STT or SOT. Two distinct behaviors are observed in Fig. 5(b). First, the DW velocity in the creep regime decreases with increasing *N* for a fixed external field, which is opposite to that in flow regime. This may be due to the increase of pinning for large *N*, which was evidenced by larger depinning field for higher *N*, in Fig. 1(d), and it has been already reported that the pinning site density is linear increased with *N*^22^. The opposite trend of DW velocity with *N* between in flow and creep regime highlights that definitely different mechanism governs the DW dynamics in each regime. Second, the similar spin current dependency is observed in the creep motion experiments. When *N* = 9, the DW moves faster for −*J*_DC_. On the other hand, in the case of *N* = 3, the DW motion for +*J*_DC_ is faster. These results imply that the SHE is dominant in thin layer, whereas the STT is more important in thicker layer. Thus, the creep motion experiments are another strong evidence to support the scenario: The SHE is inversely proportional to *N* or *t*_*FM*_, while the STT is constant for a given current density. For the case of *N* = 6, we clearly observed that the DW motion in the creep regime prefers the electron flow direction. This result, together with the DW velocity in the flow regime (for *N* = 6 in Fig. 2), suggests that the relative strength of STT and SOT can be varied depending on the dynamic regime of DW. Therefore, it should be more careful to quantify the SOT and STT from the DW motion experiments.

## Conclusions

We investigated the DW motion in two extreme regimes of DW flow and creep motions for the amorphous PMA multilayer with heavy metals, Ta/Pt/[CoSiB/Pt]_N_ nanowire structure, for different *N* and *w*. The field-driven DW velocity in the flow regime was found to increase with *N*, which is ascribed to the enhancement of DW anisotropy energy with *N*. The DW motion under a constant bias current reveals that the DW motion prefers the current flow direction in thinner layer whereas the DW motion prefers the electron flow direction in thicker layer, implying that the SHE gradually decreases with increasing the layer thickness while the STT is constant. We also found that the relative strength of two torques is different depending on the dynamic regime of DW.

## Methods

### DW flow regime

We first saturated the magnetization of the nanowire by applying sufficiently high out-of-plane magnetic field (+*H*_*z*_ = 4 kOe) and then, applied −*H*_*z*_ (*H*_*dep*_ < *H* < *H*_*C*_) to the opposite direction. Next, a pulsed current was injected into the electrode A, which creates a DW by generating a local Oersted field. Just after the creation of DW, the DW immediately moves because the applied magnetic field −*H* is already enough to push the DW to the Hall bar position. The change in Hall voltage was directly measured by oscilloscope via 46 dB amplifier. Then, now we can measure the time interval Δ*t* while the DW is moving the distance *L*, and obtain the DW velocity *v* = *L*/Δ*t*. The DW velocity was obtained by averaging the measurements ~100 times in order to get a sufficient signal to noise ratio.

### DW creep regime

Just after the creation of DW, we applied a constant magnetic field which is smaller than *H*_*dep*_. The DW arrival time *t* was measured at the Hall cross by monitoring the Hall resistance. The *t* values ranged from 0.1 to 300 s were chosen to calculate the DW velocity. Before we performed the full-scale experiments, we investigated the stochastic behavior of DW motion to make sure the creep regime. The DW arrival time was measured at a fixed magnetic field of 314 Oe for *N* = 3 and *w* = 150 nm. A clear log-normal distribution manifests that the observed DW velocity belongs to the creep regime. We repeated the measurement more than 500 times for each magnetic field. In the creep regime, we calculated the DW velocity by measuring the averaged DW arrival time *t* for the fixed wire length *L* = 50 μm at a constant bias current density of *J*_*DC*_ = ±5.4 × 10^11^ A/m^2^.

## Additional Information

**How to cite this article**: Choi, Y. H. *et al.* Field-driven domain wall motion under a bias current in the creep and flow regimes in Pt/[CoSiB/Pt]_*N*_ nanowires. *Sci. Rep.* **6**, 23933; doi: 10.1038/srep23933 (2016).

## Supplementary Material

Supplementary Information
