# Supplementary material for: Field-driven domain wall motion under a bias current in the creep and flow regimes in Pt/[CoSiB/Pt]N nanowires
Source: Sci Rep. 2016 Mar 31;6:23933. doi: 10.1038/srep23933 (PMC4814914; doi:10.1038/srep23933)
Supplement: Supplementary Information [file srep23933-s1.doc]

**Supplementary Information**

**Field-driven domain wall motion under a bias current in the creep and flow regimes in Pt/[CoSiB/Pt]*_N_* nanowires**

Y. H. Choi,^1^ Y. Yoshimura,^2^ K.-J. Kim,^2^ K. Lee,^3^ T. W. Kim,^4^ T. Ono,^2^ C.-Y. You,^5^ and M. H. Jung^1,*^

*^1^Department of Physics, Sogang University, Seoul 121-742 Korea*

*^2^Institute for Chemical Research, Kyoto University, Uji, Kyoto 611-0011, Japan*

*^3^1Institute of Physics, Johannes Gutenberg-Universität Mainz, 55128 Mainz, Germany*

*^4^Department of Advanced Materials Engineering, Sejong University, Seoul 143-747 Korea*

*^5^Department of Physics, Inha University, Incheon 402-751, Korea*

*Correspondence and requests for materials should be addressed to M.-H. J. (e-mail: mhjung@sogang.ac.kr).

1. **DW velocity characterization in the flow regime**

A typical trace of Hall signal is shown in Fig. S1(a), for the case of *N* = 3 at 950 Oe. We plot the averaged DW velocity versus magnetic field measured for different magnitudes of current density *J* = 0.5, 2.2, 2.7 and 5.4 × 10^11^ A/m^2^ in Fig. S1(b). Although the applied magnetic field was negative, we describe the field as an absolute value ignoring the sign. There is no significant change of *v* up to *J* = 2.7 × 10^11^ A/m^2^, but significant change is observed at ±*J_DC_* = 5.4 × 10^11^ A/m^2^. Therefore, we used ±*J_DC_* for the DW motion measurements with the magnetic field. Furthermore, we checked the Joule heating effect in our DW experiments, which is negligible in DC current applied up to 4 × 10^11^ A/m^2^.

1. **DW velocity characterization in the creep regime**

Before we performed the full-scale experiments, we investigated the stochastic behavior of DW motion to make sure its validity. Since the DW creep motion is thermally activated process, it should be fitted by the Arrhenius exponential law, *t*  exp(−*E_B_*/*k_B_T*), where *E_B_* is the energy barrier and *k_B_T* is the thermal energy. We repeated the measurements more than 500 times for each fixed magnetic field. The variations of $\ln\left(t\right)$ are shown in Fig. S2(a) and S2(b) for the external field of 314 and 335 Oe, respectively. Although we only display the data of two specific applied magnetic fields, these results imply that the *E_B_* shows the normal distribution and the determined *t* values are valid for all samples.

1. **Calculated DW velocity based on one dimensional model**

In this section, we calculated the DW velocity for various *N* based on Eqs. (1)-(3) of main text. To this end, we used effective anisotropy constant *K*_1_^eff^ = 1.5 × 10^5^ J/m^3^ and the saturation magnetization *M_S_* = 3.5×10^5^ A/m, which are experimentally determined. The DW anisotropy field was estimated by micromagnetic simulation as described in the main text. Walker field was calculated by considering the DW anisotropy and experimentally determined DMI. The Gilbert damping parameter was assumed to be 0.5. Figure S3 shows the calculated DW velocity for various *N* (the calculated field range is set to the same as the experimental result for +*J*). The DW velocity is found to increase with *N* in our experimental range because the DW motion lies in the intermediated regime. Since the *H*_W_ increases with *N* (*H*_W_ = 174 Oe, 262 Oe, 337 Oe for N=3, 6, 9 respectively), the effect of intermediate regime becomes more dominant with increasing *N*. The result clearly shows that the DW motion is now in the intermediate regime and the turbulent motion is not yet established in our experimental regime (especially for *N* = 9).

1. **Effect of spin Hall (SH) torque on the precessional DW motion**

As we described in the main text, SH torque rotates the magnetic moment from its stable direction which is determined by the DW anisotropy and DMI. Then the restoring field immediately rotates the magnetic moment which causes a DW motion. Although this simple explanation well describes the DW motion in a steady regime, we should be more careful when we describe the precessional DW motion, especially in two dimension (2D). For the precessional DW motion in 2D, the DW is known to be composed of vertical Bloch lines (VBLs). Such a VBL has four-fold degeneracy depending on its charge *Q* and chirality *C* as shown in Fig. S4. Note that the center of VBL has a Néel type configuration whose direction of magnetic moment is determined by the combination of *Q* and *C*; (*Q*,*C*) = (+1,+1), (-1,-1) and (*Q*,*C*) = (+1,-1), (-1,+1) have an opposite direction of magnetic moment at the center of VBL. Hence, the SH torque either assists or hinders the DW motion depending on (*Q*,*C*). Here, it is important to note that the average DW velocity is not affected by the SH torque since the four VBLs appears with equal probability because they are energetically equivalent.

It is recently reported that the DMI lifts the degeneracy of VBLs and splits their energy levels into two sub-levels, i.e., ground and excites states [ref. yoshimura arxiv]. Figure S4 shows this situation. Since the width of VBL depends on the DW anisotropy and DMI, the DMI can modulate the width of VBL; the width of the ground state VBL is larger than that of the excited VBL. Due to this symmetry breaking, the SH torque can be effective. That is to say, if DMI exists, the average SH torque is not averaged out and affects the DW motion. This is the physical background of the effect of SH torque on the precessional DW motion in 2D.

1. **Threshold current density and the effect of Joule heating.**

In order to estimate the threshold current density *J*_C_, we performed a magnetic DW depinning measurement. We first created a DW in the nanowire and applied magnetic field to depin the DW. Then the DW depinning field *H*_d_ is obtained with various bias current densities. Figure S5 shows the DW depinning field *H*_d_ and *H*_C_ as a function of bias current density *J* for *N*=6. The trend of *H*_d_ comprises linear and quadratic components. The linear component arises from the effect of spin transfer torque as we described in our manuscript, whereas the quadratic component may originate from the current-induced Joule heating. Since *J*_C_ is defined as a threshold current density that can move the DW *in the absence of* magnetic field, *J*_C_ can be determined by extracting the value of *x*-intersection. As shown in Fig. S5, we did not observe the current-only-driven DW motion in our experimental range; that is, *J*_C_ is found to be bigger than 1.3 × 10^12^ A/m^2^. This clearly indicates that the applied *J*_DC_ (= 5.4 × 10^11^ A/m^2^) is much smaller than the critical current density *J*_C_. In this sense, we described that the DW motion as current-assisted field-driven DW motion.

Current-induced Joule heating effect affects the magnetic properties. Such an effect can be readily checked by current-dependent coercive field *H*_C_, since *H*_C_ depends on the magnetic anisotropy *K*_U_ and the saturation magnetization *M*_S_. As seen in Fig. S5, *H*_C_ is found to be almost constant up to |*J*| = |*J*_DC_| = 5 × 10^11^ A/m^2^, above which significant decrease of *H*_C_ is observed due to the Joule heating. This demonstrates that *J*_DC_ used in our experiment does not cause a significant Joule heating.

**Figure S1** (a) Hall voltage *V_H_* versus arrival time *t* at the Hall cross bar of [CoSiB/Pt]_N_ nanowires for *N* = 3 with *J_DC_* = -5.4 × 10^11^ A/m^2^ in *H* = 950 Oe. Vertical red lines indicate the starting and arrival time of DW. (b) DW velocity *v* versus magnetic field *H* for *N* = 3 with various current densities. The data were plotted by averaging the measurements ~100 times.

**Figure S2** Distribution of DW arrival time in the creep regime at (a) 314 Oe and (b) 335 Oe for [CoSiB/Pt]_N_ nanowires with *N* = 3 and *w* = 150 nm. Total number of repeated measurements is more than 500 for each magnetic field.


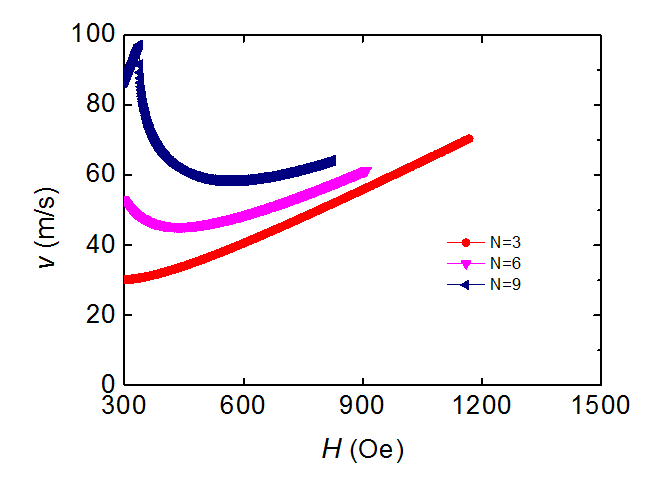


**Figure S3** Calculated DW velocity as a function of magnetic field *H* for various *N*.


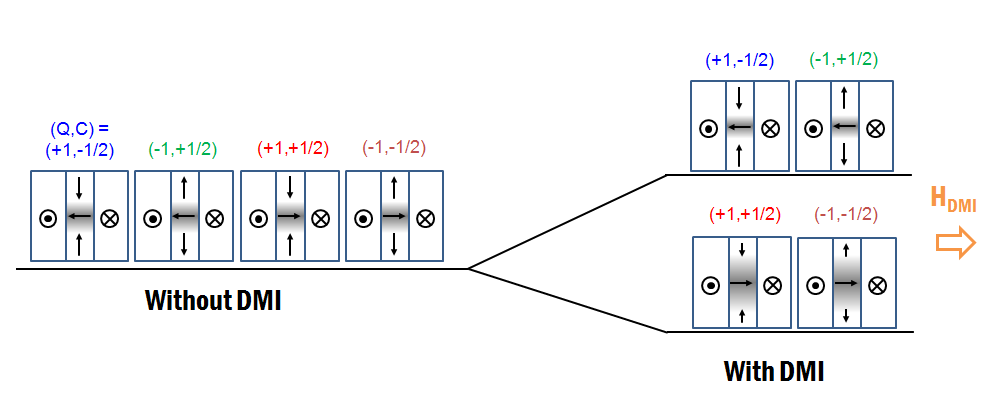


**Figure S4** vertical Bloch lines (VBLs) and energy splitting of VBL induced by the DMI.

**Figure S5**. *J*_DC_ dependence of *H*_dep_ and *H*_C_ for N = 6.
